# Association of IL-1*β*, NLRP3, and COX-2 Gene Polymorphisms with Autoimmune Thyroid Disease Risk and Clinical Features in the Iranian Population

**DOI:** 10.1155/2021/7729238

**Published:** 2021-11-08

**Authors:** Zahra Heidari, Saeedeh Salimi, Mohsen Rokni, Mahnaz Rezaei, Neshat Khalafi, Mahdieh Jafari Shahroudi, Azizallah Dehghan, Mohsen Saravani

**Affiliations:** ^1^Department of Endocrinology and Metabolism, Zahedan University of Medical Sciences, Zahedan, Iran; ^2^Department of Clinical Biochemistry, School of Medicine, Zahedan University of Medical Sciences, Zahedan, Iran; ^3^Cellular and Molecular Research Center, Resistant Tuberculosis Institute, Zahedan University of Medical Sciences, Zahedan, Iran; ^4^Department of Immunology, School of Medicine, Tehran University of Medical Sciences, Tehran, Iran; ^5^Department of Nutrition, School of Medicine, Zahedan University of Medical Sciences, Zahedan, Iran; ^6^Noncommunicable Diseases Research Center, Fasa University of Medical Sciences, Fasa, Iran

## Abstract

**Background:**

Grave's disease (GD) and Hashimoto's thyroiditis (HT) are autoimmune diseases of the thyroid gland in which genetic predisposition plays a major role in their development. Currently, the role of NLRP3 inflammasome and COX-2 has been documented in many autoimmune diseases. The purpose of the study is to delineate the impact of IL-1*β* (rs1143634), NLRP3 (rs3806265), and COX-2 (rs2745557) gene polymorphisms in the development of GD and HT.

**Methods:**

A total of 256 newly diagnosed patients with autoimmune thyroid disease (135 patients with HT and 121 GD patients) as case groups and 145 controls were included in the study.

**Results:**

Recessive and overdominant models showed a significant association between IL-1*β* rs1143634 SNP and HT development risk. The frequency of TT genotype and T allele of IL-1*β* rs1143634 SNP in the control group was significantly higher than the GD group. There was no significant association between NLRP3 rs3806265 polymorphism and HT and GD development. The frequency of GA genotype of COX-2 (rs2745557) in the control group was significantly higher than that in the HT group. There was no significant association between COX-2 rs2745557 genotypic and allelic distribution and GD development risk. The results revealed a significant relationship between some clinical features of HT and GD groups and SNPs studied.

**Conclusion:**

The results manifest the significant impact of IL-1*β* rs1143634 and COX-2 (rs2745557) SNPs and HT development and IL-1*β* rs1143634 SNP on GD occurrence risk. Furthermore, a significant relationship was observed between some clinical features of HT and GD groups and studied SNPs.

## 1. Introduction

Autoimmune thyroid disease (AITD) can occur as a part of autoimmune diseases with either hyperthyroidism manifestations (GD) or hypothyroidism (HD, also known as chronic lymphocytic thyroiditis) [1]. The prevalence of AITD was about 7–8% in the general population [2] and about 4-10 times higher in inwomen than men [3]. The general incidence of GD has been reported approximately 24.8 cases per 100000. HT is a most common autoimmune disease with a prevalence of 10–12% in the general population [1, 4]. Delshad et al. showed 0.4% and 0.8% frequency of clinical hypothyroidism in men and women and 0.1% and 0.2% of clinical hyperthyroidism in men and women in a sample of the Iranian population in Tehran province, respectively [5]. AITD is a multifactorial disease in which genetic predisposition plays a major role [6, 7]; however, its exact molecular mechanism is still unclear and needs further research [8]. Some factors such as contact with radiation, vitamin D and selenium deficiency, viral infections, and excess iodine in the diet are environmental factors for AITD development [9]. Furthermore, some genes such as protein tyrosine phosphatase, human leukocyte antigen/HLA-DR, and thyroid-specific genes (TG and TSH) are candidate genes linking the genetic factors to AITD risk [10].

The pathogenesis of AITD is characterized by increased lymphocyte production of inflammatory cytokines such as interleukin-1*β* (IL-1*β*), interferon-gamma (IFN-*γ*), and tumor necrosis factor-alpha (TNF*α*). The activation of IL-1 *β* is mediated by certain intracellular multiprotein plats called; inflammasome is comprised of three main components: the adaptor protein called ASC (apoptosis-associated speck-like protein containing a CARD), initiator protein like pattern recognition receptors (PRRs), and the effector protein, pro-caspase-1 [11]. Activated inflammasome, as a multiprotein complex, binds to pro-caspase-1 via intermediate action of the adaptor molecule apoptosis-associated speck-like protein containing a CARD (ASC), leading to conversion of pro-interleukin-1*β* (pro-IL-1*β*) and pro-interleukin-18 (pro-IL-18) into mature IL-1*β* and IL-18 [12]. Furthermore, the activation of the inflammasome also activates a kind of programmed cell death named pyroptosis [13]. NLRP3 inflammasome is stimulated by several kinds of stimulators such as RNA viruses and toxins [14]. Prostaglandins (PGs), the product of the cyclooxygenase (COX) pathway, have definite roles in the immune response and inflammation of autoimmune diseases. COX has two isoforms called COX-1 with permanent expression in most tissues and COX-2 induced at the site of inflammation; COX-2 seems to play a major function in the development of inflammation and autoimmune diseases [15].

Currently, the role of NLRP3 inflammasome and COX-2 has been documented in autoimmune diseases such as rheumatoid arthritis (RA) and systemic lupus erythematosus (SLE) [15, 16]. Many studies have reported that thyroid follicular cells (TFC) express toll-like receptors (TLR) such as TLR 4, 7, and 9 which respond to various pathogen-associated molecular patterns (PAMPs) and endogenous damage-associated molecular patterns (DAMPs) including uric acid crystals, aluminum hydroxide crystals used in vaccine adjuvants, adenosine triphosphate (ATP) released from mitochondria, silica, bacterial products, bacterial toxins produced by streptococci and staphylococci, bacterial DNA-RNA hybrids, and the influenza virus, to induce the activation of caspase-1, and also the main function of caspase-1 is to convert the inactive forms of IL-1*β* and IL-18 to active forms, which leave the cell and perform various proinflammatory functions [17–19]. This process leads to the chemotaxis of the innate immune system and self-reactive lymphocytes to the thyroid, and then the production of multiple proinflammatory cytokines by lymphocytes leads to injury or apoptosis in thyroid follicular cells (TFCs) and contributes to the immune pathogenesis of AITD [17]. Recently, D'Ascola et al. provided in vitro evidence that supports the role of Toll-like receptors (TLR-2 and TLR-4) in driving the inflammatory response in thyrocytes and consequent damages and loss of function by reducing the expression of thyroid-specific genes [20].

The results of a study conducted in 2018 suggested a feedback loop mechanism in which inflammasome leads to immune cell activation (e.g., secretion IL-1*β* and IL-18); this mechanism may play important roles in the cascade of follicular destruction and lymphatic recruitment in the thyroid of autoimmune thyroiditis patients [21]. More recent studies indicate that NLRP3 variations may be associated with several autoimmune diseases including neonatal-onset multisystem inflammatory disease (NOMID) and Behcet's disease (BD) [22].

Single nucleotide polymorphisms (SNPs) are point mutations that occur in at least one percent of the population, which might underlie differences in susceptibility to diseases, response to treatments, and the severity of illness [23]. Polymorphism in IL-1*β* was found to be related to some autoimmune and infection diseases such as periodontitis and tuberculosis [24].

Studies have proposed the relationship between IL-1, NLRP3, and COX-2 SNPs and autoimmune diseases such as systemic lupus erythematosus (SLE) and autoimmune thyroid disease [15, 25–27]. Accordingly, the aim of this study was to define the relationship of IL-1*β* +3954 C/T (rs1143634), NLRP3 (rs3806265) T/C, and COX-2 (rs2745557) G/A gene polymorphisms susceptible to autoimmune thyroiditis development.

## 2. Subjects and Methods

### 2.1. Study Subjects

A total of 256 newly diagnosed patients with autoimmune thyroid disease (135 HT patients and 121 GD patients) as case groups and 145 age- and gender-matched controls were included in the study. Inclusion and exclusion criteria were AITD patients referring to Ali-ebn Abitaleb Hospital, Zahedan, South-East Iran, were diagnosed by an endocrinologist based on laboratory profile and clinical manifestations. Patients with other autoimmune diseases except AITD were excluded from the study. Controls were selected from healthy individuals who came to the same hospital for a checkup. Controls with a history of autoimmune diseases and family relationships with patients with autoimmune disorders were excluded from the study. The study protocol was approved by the Ethics Committee of the Zahedan University of Medical Sciences (IR.ZAUMS.REC.1398.206); also, written informed consent was obtained from all participants.

### 2.2. DNA Extraction and Genotype Analysis

Genomic DNA was extracted from EDTA-contained venous blood according to the salting-out protocol [28]. The quantity and quality of DNA were confirmed by Nanodrop, and DNA was stored at -20°C. The IL-1*β* rs1143634, NLRP3 rs3806265, and COX-2 rs2745557 were genotyped by restriction fragment length polymorphism (RFLP) method.  Table 1 shows the pairs of PCR primer sequences and restriction enzyme for each DNA product [22, 24, 29]. The optimized PCR conditions included 2 min of denaturation at 95°C followed by 30 cycles of denaturation at 95°C for 30 s, annealing at 54°C for 30 s for IL-1*β*, at 54°C for 30 s for NLRP3 or at 59°C for 30 s for COX-2, and extension at 72°C for 30 s. The PCR was completed with a final extension step of 3 min at 72°C. All of the gene products were digested overnight at the enzyme optimal temperature as the digested fragments were separated in a 2% agarose gel.

### 2.3. Statistical Analysis

Statistical analysis was performed by SPSS version 23.0. The categorical and continuous variables were analyzed using the *χ*^2^ and independent sample *t*-test, respectively. The regression logistic method was used to evaluate the effects of each polymorphism and haplotype on the risk of developing HT and GD. *P* value < 0.05 was considered statistically significant.

## 3. Results

### 3.1. Demographic and Clinical Characteristics of HT and GD Patients

There was no statistically significant difference between controls and patients in both HT and GD as regards age, with *P* = 0.196 and *P* = 0.774, respectively, or gender distribution with *P* = 0.072 and *P* = 0.092, respectively. Tables 2 and 3 show demographic and clinical characteristics of HT and GD patients, respectively.

### 3.2. SNP's Genotypes and Allelic Distribution and HT Development Risk

Table 4 shows the genotypic and allelic distribution of IL-1*β*, NLRP3, and COX-2 genes SNPs in the HT and control groups. There was no significant association between IL-1*β* rs1143634 genotypic and allelic distribution and HT development risk. However, recessive (CC + CT vs. TT) and overdominant (CC + TT vs. CT) models showed a significant association between IL-1*β* rs1143634 SNP and HT development risk so may be protective and risk factors against the development of HT, respectively (*P* = 0.03, OR = 0.67, 95%CI = 0.21 − 0.91; *P* = 0.03, OR = 1.1, 95%CI = 0.76 − 1.8, respectively).

In addition, there was no significant association between NLRP3 rs3806265 genotypic and allelic distribution and HT development risk. Similar results were also found regarding the genetic models (dominant, recessive, and overdominant models).

As regard COX-2 (rs2745557), the frequency of GA genotype was significantly higher in the control group than that in the HT group and may be a protective factor for HT development (*P* = 0.017, OR = 0.53, 95%CI = 0.31 − 0.89) (Table 4). Similarly, the dominant (GG vs. GA + AA) and overdominant (GG + AA vs. GA) models showed the same results (*P* = 0.035, OR = 0.56, 95%CI = 0.36 − 0.96; *P* = 0.015, OR = 0.53, 95%CI = 0.31 − 0.89, respectively). The allelic frequency showed no significant difference between the two groups (*P* = 0.73).

### 3.3. SNP's Genotypes and Allelic Distribution and GD Development Risk

Table 5 shows the genotypic and allelic distribution of IL-1*β*, NLRP3, and COX-2 genes SNPs in GD and control groups. As regard IL-1 *β* gene, the frequency of TT genotype in the control group was significantly higher than that in the GD group and may be a protective factor for GD development (*P* = 0.002, OR = 0.24, 95%CI = 0.097 − 0.6). Similarly, the recessive (CC + CT vs. TT) model showed the same results and may be a protective factor for GD development (*P* = 0.002, OR = 0.26, 95%CI = 0.11 − 0.6). The frequency of T allele in the control group was significantly higher than that in the GD group and may act as a protective factor for GD development (*P* = 0.009, OR = 0.61, 95%CI = 0.43 − 0.88).

There was no significant association between NLRP3 rs3806265 genotypic and allelic distribution and GD development risk. Same results were also found regarding the genetic models (dominant, recessive, and overdominant models) and allelic distribution.

There was no significant association between COX-2 rs2745557 genotypic and allelic distribution and GD development risk (Table 5). Same results were also found regarding the genetic models (dominant, recessive, and overdominant models) and allelic distribution.

### 3.4. Association between Genes SNPs and Clinical Features of HT and GD Groups

In the HT group, the level of the anti-TPO in the CT genotype of the NLRP3 SNP was higher than the CC genotype (576.7 ± 64.4 vs. 270.2 ± 50.8, respectively, *P* = 0.011) (Table 6). Regarding the age of onset, there was a significant association between IL-1*β* SNP CT and TT genotypes (*P* = 0.011). In case of the family history, there was a significant association with IL-1*β* SNP (*P* = 0.033).

In the GD group, the levels of FT3 and FT4 in the CC genotype of the NLRP3 SNP were lower than the TT genotype (*P* = 0.001) (Table 6).

Regarding COX-2 SNP, the level of TSH level was higher in the GA genotype than in the GG genotype in the GD group (0.025 ± 0.005 vs. 0.0105 ± 0.002, respectively, *P* = 0.019). The TSH level in CT and CC genotypes of the IL-1*β* SNP was lower than TT genotype in the GD group (*P* = 0.008, *P* = 0.045, respectively).

## 4. Discussion

The current study concluded that as regard HT, the recessive and overdominant models of IL-1*β* rs1143634 SNP as well as the GA genotype and dominant and overdominant models of COX-2 rs2745557 may be protective from disease development. However, no significant association was found between NLRP3 rs3806265 SNP and HT.

As regard GD, both T allele, TT genotype, and the recessive model of IL-1*β* rs114364 SNP may be protective from GD. However, no significant association was found between NLRP3 rs3806265 and COX-2 rs2745557 SNPs.

In addition, IL-1*β* SNPs and COX-2 showed a significant association with TSH in the GD group. Also, NLRP3 SNP was significantly associated with FT3 and FT4 levels in the GD group. In HT group, NLRP3 and IL-1*β* SNPs was significantly associated with anti-TPO level and with age of onset, respectively.

Autoimmune thyroid diseases (AITDs) as one of the most organ-specific autoimmune diseases are more common in women than men [30]. Among the AITDs, both HT and GD are considered the main causes of hypothyroidism and hyperthyroidism, respectively [31]. Studies have confirmed the link between environmental and genetic factors for AITD development [7]. SNPs are the most common studied genetic element responsible for interindividual differences in disease susceptibility, severity of illness, and response to therapy [23].

Experimental studies have shown that IL-1*β* could stimulate the thyroid follicular cells to produce cytokines which increase the inflammatory response in AITD via nitric oxide (NO) and prostaglandin (PG) production. Pathogenic action of IL-1*β* in thyroid ophthalmopathy was also reported. IL-1*β* effect on goitre development occurs through stimulation of hyaluronic acid production in thyroid epithelial cells and fibroblasts [32]. IL-1*β* influences thyroid function via stimulation of secretion of inflammatory cytokines such as IL-6. Finally, IL-1*β* could induce apoptosis and tissue damage in thyroid follicular cells. A protective effect of TT genotype, and T allele of IL-1*β* rs1143634 SNP was found on GD development. Similar results were also found about recessive and overdominant models and HT risk [33]. IL-1*β* rs1143634 (+3594) polymorphism, as a coding synonymous variant, was mapped to exon 5 of the IL-1*β* gene. The substitution of C with T does not change amino acid coding which leads to the production of a truncated protein that is likely to be rapidly degraded or functionally inactive. Pociot et al. reported that the T allele of the IL − 1*β* + 3954 SNP increases IL-1*β* production in response to LPS [34, 35]. The findings are in agreement with the study of Zaaber et al., suggesting a significant relation between IL-1*β* rs1143634 SNP and GD and HT in the Tunisian population [27]. Lacka et al. and Rashad et al. also found a significant relationship between IL-1*β* rs1143634 SNP and HT in a Polish and Egyptian population, respectively [36, 37]. In contrast, Chen et al. found no association between IL-1*β* rs1143634 SNP and GD risk in Taiwan [38], while Liu et al. found a positive relationship between the C allele of rs1143634 and GD risk [39]. In a meta-analysis, Wong et al. reported no significant association of L1-B rs1143634 SNP and Graves' ophthalmopathy [40].

Liu et al. demonstrated that excessive iodine intake, as a mechanism for HT development, leads to an increase of pyroptosis in thyroid follicular cells (TFCs) via NF-*κ*B, NLRP3, and IL-1*β* pathway [41]. Guo et al. revealed the upregulation of some cytokines such as NLRP3, caspase-1, and pro IL-1*β* in HT tissue accompanied by posttranslational maturation of caspase-1 and IL-1*β* [21]. The our results showed no significant association between NLRP3 rs3806265 SNP and HT and GD development in the Iranian population. However, there is evidence suggesting NLRP3 SNP as a dispose factor for several autoimmune diseases. In a meta-analysis, Zhang et al. found an increase in susceptibility to rheumatoid arthritis, inflammatory bowel disease, and ulcerative colitis [42]. Yu et al. also showed a significant association of NLRP3 rs3806265 SNP with psoriasis vulgaris in the Chinese Han population [43]. Sharon and Jiquan demonstrated a significant correlation of NLRP1 and NLRP3 gene polymorphism with psoriasis [44]. To the best of our knowledge, this study is the first evaluation between NLRP3 gene polymorphism and AITD. However, no association was detected between them. More studies on different ethnicities and sample sizes are necessary to evaluate the impact of NLRP3 gene polymorphism on AITD. A possible mechanism is diversity in the human population that may cause variation in genotypic and allelic frequencies.

The information about COX-2 expression in normal and autoimmune thyroid tissues is contradictory. Some studies have reported the detectable expression of COX-2 in normal thyroid [45], while some data have emphasized that COX-2 proteins are not expressed in normal thyroid tissues. Immunohistochemical studies have shown a high level of COX-2 in HT tissue [46]. Fuhrer et al. revealed a similar mRNA level of COX-2 in normal thyroid and Graves' samples [47] although an immunohistochemical study showed negative COX-2 staining in Graves' disease and positive COX-2 staining in Hashimoto disease [48]. Based on the our finding, COX-2 rs2745557 SNP proposed as a protective factor for HT development, while no association of COX-2 rs2745557 SNP with GD development was observed. Recently, no significant association of COX-2 rs2745557 SNP with SLE development has been reported [15]. In a case-control study, Lee et al. showed no significant effect of COX-2 -765G/C SNP on rheumatoid arthritis (RA) development risk and severity in a Korean population [49]. In another case-control study performed on a Korean population, Yun et al. showed no significant association of COX − 2 − 1329A > G SNP and the risk of RA development; however, they found a significant impact of 6365 T > C and −899G > C SNPs on RA development risk as a protective factor. Their results also showed no association between COX-2 SNP and the radiologic severity of RA [50]. To the best of our knowledge, this is the first study to investigate the relation between COX-2 SNP and thyroid autoimmune diseases although Uçan et al. conducted a study to evaluate the association of COX-2 SNP with differentiated thyroid carcinomas and found a significant correlation between COX-2 SNP and differentiated thyroid carcinomas [51].

The results revealed a significant association between anti-TPO and NLRP3 SNP, the family history, and the age of onset with IL-1*β* SNP in the HT group. Furthermore, a significant relationship was found between the levels of FT3 and F4 levels with NLRP3 SNP, the TSH level with COX-2, and IL-1*β* SNPs. Krawczyk-Rusiecka et al. reported a relation between COX-2 expression and anti-TPO antibodies levels in the HT group [46]. Guo et al. demonstrated a significant association between the NLRP1 mRNA level and serum TgAb and TPOAb levels in autoimmune thyroid patients [21].

The present study demonstrated a significant association between IL-1*β* (rs1143634) and COX-2 (rs2745557) SNPs and HT development. Furthermore, IL-1*β* (rs1143634) SNP significantly affected GD development. Moreover, a significant relationship was detected between some clinical features of HT and GD groups and SNPs studied. A limitation of this study is its relatively small sample size. Further studies are required to validate the findings in other ethnic groups.

## Figures and Tables

**Table 1:** Primers and restriction enzymes used for RFLP-PCR method.

Gene and SNP number	Primers	Restriction enzyme	DNA fragment size (bp)
IL-1*β* (rs1143634)	F: GTTGTCATCAGACTTTGACC R: TTCAGTTCATATGGACCAGA	Taq I	TT = 249 CC = 135 + 114
COX-2 (rs2745557)	F: GAGGTGAGAGTGTCTCAGAT R: TCTCGGTTAGCGACCAATT	Taq I	GG = 439 AA = 353 + 76
NLRP3 (rs3806265)	F: TTGGCAGGTGGACAGCAGCA R: GACCCCAAACATCCCCCAAATCA	Pvu II	TT = 127 CC = 105 + 22

F: forward; R: reverse.

**Table 2:** Demographic and clinical characteristics of Hashimoto (HT) patients and controls.

	HT *n* = 131	Control *n* = 145	*P* value
Age, years	33.99 ± 11.53	35.77 ± 11.25	0.196
Gender			
Male (%)	10 (7.6)	21 (14.48)	0.072
Female (%)	121 (92.4)	124 (85.52)	
BMI (kg/m^2^)	26.8 ± 5.8		
Onset age	31.36 ± 10.7		
Other autoimmune disorders history (%)	2 (1.52)		
Family history (%)	41 (31.29)		
Smoking history (%)	11 (8.4)		
Thyroid size (ml)	8.21 ± 0.52		
Free T4 (ng/dl + SEM)	0.477 ± 0.013		
Free T3 (pg/ml ± SEM)	1.6 ± 0.033		
TSH (mU/L ± SEM)	62.38 ± 2.5		
Thyroid peroxidase antibody (TPOAb) (IU/ml ± SEM)	453 ± 41.76		
Antithyroglobulin antibody (IU/ml ± SEM)	732 ± 122		

**Table 3:** Demographic and clinical characteristics of Graves' patients and controls.

	Graves' *n* = 125	Control *n* = 145	*P* value
Age, years	35.37 ± 11.88	35.77 ± 11.25	0.774
Gender			
Male (%)	28 (22.4)	21 (14.48)	0.092
Female (%)	97 (77.6)	124 (85.52)	
BMI	23.06 ± 0.43		
Onset age	34.38 ± 1.06		
Other autoimmune disorders history (%)	1 (0.8)		
Family history (%)	40 (32)		
Smoking history (%)	20 (16)		
Thyroid volume (ml)	21.8 ± 1.53		
Free T4 (ng/dl + SEM)	3.14 ± 1.04		
Free T3 (pg/ml ± SEM)	6.64 ± 0.18		
TSH (mU/L ± SEM)	0.016 ± 0.002		
Graves ophthalmopathy	28 (22.4)		

**Table 4:** Allelic and genotypic frequency of NLRP3, COX-2, and IL-1*β* polymorphisms in Hashimoto's thyroiditis (HT) and control groups.

Polymorphism	HT (*n* (%))	Control (*n* (%))	*P* value^∗^	OR (95% CI)
IL-1*β* (rs1143634)				
Codominant				
CC	42 (31.1)	52 (35.9)	1	1
CT	80 (59.2)	66 (45.5)	0.131	1.5 (0.89-2.5)
TT	13 (9.7)	27 (18.6)	0.157	0.56 (0.25-1.24)
Dominant				
CC	42 (31.1)	52 (35.9)	1	1
CT + TT	93 (68.9)	93 (64.1)	0.423	1.2 (0.74-2)
Recessive				
CC + CT	123 (90.3)	125 (81.4)	1	1
TT	13 (9.7)	27 (18.6)	0.03	0.67 (0.21-0.91)
Overdominant				
CC + TT	53 (40.8)	79 (54.5)	1	1
CT	80 (59.2)	66 (45.5)	0.03	1.1 (0.76-1.8)
Allele				
C	164 (60.7)	170 (58.6)	1	1
T	106 (39.3)	120 (41.4)	0.389	0.86 (0.6-1.2)
NLRP3 (rs3806265)				
Codominant				
TT	45 (33.3)	59 (40.7)	1	1
CT	61 (45.2)	59 (40.7)	0.279	1.3 (0.78 –2.2)
CC	29 (21.5)	27 (18.6)	0.325	1.4 (0.72-2.5)
Dominant				
TT	45 (33.3)	59 (40.7)	1	1
CT + CC	90 (66.7)	86 (59.3)	0.224	1.35 (0.83-2.2)
Recessive				
TT + CT	106 (78.5)	118 (81.4)	1	1
CC	29 (21.5)	27 (18.6)	0.568	1.2 (0.65-2.1)
Overdominant				
TT + CC	74 (54.8)	86 (59.3)	1	1
CT	61 (45.2)	59 (40.7)	0.466	1.2 (0.74-1.9)
Allele				
T	151 (55.9)	177 (61)	1	1
C	119 (44.1)	113 (39)	0.26	1.2 (0.87-1.7)
COX-2 (rs2745557)				
Codominant				
GG	88 (65.2)	77 (53.1)	1	1
GA	36 (26.6)	59 (40.6)	0.017	0.53 (0.31-0.89)
AA	11 (8.2)	9 (6.3)	0.991	0.99 (0.38-2.5)
Dominant				
GG	88 (65.2)	77 (53.1)	1	1
GA + AA	47 (34.8)	68 (46.9)	0.035	0.56 (0.36-0.96)
Recessive				
GG + GA	124 (91.8)	136 (93.7)	1	1
AA	11 (8.2)	9 (6.3)	0.641	1.24 (0.49-3.1)
Overdominant				
GG + AA	99 (73.4)	66 (59.4)	1	1
GA	36 (26.6)	59 (40.6)	0.015	0.53 (0.31-0.89)
Allele				
G	212 (78.5)	213 (73.5)	1	1
A	58 (21.5)	77 (26.5)	0.73	0.49 (0.31-0.88)

**Table 5:** Allelic and genotypic frequency of NLRP3, COX-2, and IL-1*β* polymorphisms in Graves' disease (GD) and control groups.

Polymorphism	GD (*n* (%))	Control (*n* (%))	*P* value^∗^	OR (95% CI)
IL-1*β* (rs1143634)				
Codominant				
CC	56 (44.8)	52 (35.9)	1	1
CT	62 (49.6)	66 (45.5)	0.601	0.87 (0.52-1.45)
TT	7 (5.6)	27 (18.6)	0.002	0.24 (0.097-0.6)
Dominant				
CC	56 (44.8)	52 (35.9)	1	1
CT + TT	68 (55.2)	93 (64.1)	0.136	0.69 (0.42-1.1)
Recessive				
CC + CT	118 (94.4)	125 (81.4)	1	1
TT	7 (5.6)	27 (18.6)	0.002	0.26 (0.11-0.6)
Overdominant				
CC + TT	63 (50.4)	79 (54.5)	1	1
CT	62 (49.6)	66 (45.5)	0.503	1.1 (0.72-1.9)
Allele				
C	174 (69.6)	170 (58.6)	1	1
T	76 (30.4)	120 (41.4)	0.009	0.61 (0.43-0.88)
NLRP3 (rs3806265)				
Codominant				
TT	43 (34.4)	59 (40.7)	1	1
CT	46 (36.8)	59 (40.7)	0.81	1.07 (0.61 –1.83)
CC	36 (28.8)	27 (18.6)	0.062	1.8 (0.96-3.4)
Dominant				
TT	43 (34.4)	59 (40.7)	1	1
CT + CC	82 (65.6)	86 (59.3)	0.288	1.3 (0.8-2.1)
Recessive				
TT + CT	89 (71.2)	118 (81.4)	1	1
CC	36 (28.8)	27 (18.6)	0.05	1.7 (1-1.3)
Overdominant				
TT + CC	79 (63.2)	86 (59.3)	1	1
CT	46 (36.8)	59 (40.7)	0.513	0.85 (0.51-1.3)
Allele				
T	132 (52.8)	177 (61)	1	1
C	118 (47.2)	113 (39)	0.055	1.4 (0.99-1.9)
COX-2 (rs2745557)				
Codominant				
GG	66 (52.8)	77 (53.1)	1	1
GA	47 (37.6)	59 (40.6)	0.776	0.92 (0.56-1.5)
AA	12 (9.6)	9 (6.3)	0.349	1.55 (0.61-3.5)
Dominant				
GG	66 (52.8)	77 (53.1)	1	1
GA + AA	59 (47.2)	68 (46.9)	0.96	1 (0.62-1.6)
Recessive				
GG + GA	113 (90.4)	136 (93.7)	1	1
AA	12 (9.6)	9 (6.3)	0.303	1.6 (0.65-3.9)
Overdominant				
GG + AA	78 (62.4)	86 (59.4)	1	1
GA	47 (37.6)	59 (40.6)	0.604	0.88 (0.53-1.4)
Allele				
G	179 (71.6)	213 (73.5)	1	1
A	71 (28.4)	77 (26.5)	0.698	1 (0.75-1.6)

**Table 6:** Association of NLRP3, COX-2, and IL-1*β* SNPs with clinical and demographic characteristics of HT and GD groups.

HT group						
Mean ± SEM				Genotypes		
NLRP3 SNP	TT	CT	CC	CT vs. TT	CC vs. TT	CT vs. CC
Anti-TPO	411.9 ± 74.4	576.7 ± 64.4	270.2 ± 50.8	0.169	0.402	0.011
IL-1*β* SNP	CC	CT	TT	CT vs. CC	TT vs. CC	CT vs. TT
Age of onset	30.09 ± 1.5	33 ± 1.2	25.08 ± 2.6	0.311	0.311	0.011
GD group						
Mean ± SEM				Genotypes		
NLRP3 SNP	TT	CT	CC	CT vs. TT	CC vs. TT	CT vs. CC
FT4	3.6 ± 0.23	3.1 ± 0.13	2.63 ± 0.11	0.088	0.001	0.148
FT3	7.45 ± 0.36	6.54 ± 0.27	5.79 ± 0.27	0.087	0.001	0.209
COX-2 SNP	GG	GA	AA	GA vs. GG	AA vs. GG	GA vs. AA
TSH	0.0105 ± 0.002	0.025 ± 0.005	0.015 ± 0.002	0.019	0.901	0.106
IL-1*β* SNP	CC	CT	TT	CT vs. CC	TT vs. CC	CT vs. TT
TSH	0.01 ± 0.002	0.0176 ± 0.004	0.045 ± 0.019	0.321	0.008	0.045

## Data Availability

The data that support the findings of this study are available from the corresponding author, upon reasonable request.

## References

[1] Wiersinga W. M., Vitti P., Hegedüs L. (2018). Hashimoto’s thyroiditis. *Thyroid Diseases: Pathogenesis, Diagnosis, and Treatment*.

[2] Ramgopal S., Rathika C., Padma Malini R., Murali V., Arun K., Balakrishnan K. (2019). Critical amino acid variations in HLA-DQB1∗ molecules confers susceptibility to autoimmune thyroid disease in South India. *Genes & Immunity*.

[3] Liontiris M. I., Mazokopakis E. E. J. H. J. N. M. (2017). A concise review of Hashimoto thyroiditis (HT) and the importance of iodine, selenium, vitamin D and gluten on the autoimmunity and dietary management of HT patients. *Points that need more investigation*.

[4] Hussain Y. S., Hookham J. C., Allahabadia A., Balasubramanian S. P. (2017). Epidemiology, management and outcomes of Graves' disease-real life data. *Endocrine*.

[5] Delshad H., Mehran L., Tohidi M., Assadi M., Azizi F. (2012). The incidence of thyroid function abnormalities and natural course of subclinical thyroid disorders, Tehran, I.R. Iran. *Journal of endocrinological investigation*.

[6] Hwangbo Y., Park Y. J. (2018). Genome-wide association studies of autoimmune thyroid diseases, thyroid function, and thyroid cancer. *Endocrinology and Metabolism*.

[7] Ruggeri R. M., Giuffrida G., Campennì A. (2018). Autoimmune endocrine diseases. *Minerva endocrinologica*.

[8] Dong Y., Fu D. (2014). Autoimmune thyroid disease: mechanism, genetics and current knowledge. *European Review for Medical and Pharmacological Sciences*.

[9] Ferrari S. M., Fallahi P., Antonelli A., Benvenga S. (2017). Environmental issues in thyroid diseases.

[10] Kyritsi E. M., Kanaka-Gantenbein C. (2020). Autoimmune thyroid disease in specific genetic syndromes in childhood and adolescence.

[11] Song N., Li T. (2018). Regulation of NLRP3 inflammasome by phosphorylation. *Frontiers in immunology*.

[12] Yang Y., Wang H., Kouadir M., Song H., Shi F. (2019). Recent advances in the mechanisms of NLRP3 inflammasome activation and its inhibitors. *Cell Death & Disease*.

[13] Shi J., Zhao Y., Wang K. (2015). Cleavage of GSDMD by inflammatory caspases determines pyroptotic cell death. *Nature*.

[14] Mariathasan S., Weiss D. S., Newton K. (2006). Cryopyrin activates the inflammasome in response to toxins and ATP. *Nature*.

[15] Sandoughi M., Saravani M., Rokni M., Nora M., Mehrabani M., Dehghan A. (2020). Association between COX-2 and 15-PGDH polymorphisms and SLE susceptibility. *International Journal of Rheumatic Diseases*.

[16] Shen H.-H., Yang Y.-X., Meng X. (2018). NLRP3: a promising therapeutic target for autoimmune diseases.

[17] Kawashima A., Tanigawa K., Akama T., Yoshihara A., Ishii N., Suzuki K. (2011). Innate immune activation and thyroid autoimmunity. *The Journal of Clinical Endocrinology & Metabolism*.

[18] Harii N., Lewis C. J., Vasko V. (2005). Thyrocytes express a functional toll-like receptor 3: overexpression can be induced by viral infection and reversed by phenylmethimazole and is associated with Hashimoto’s autoimmune thyroiditis. *Molecular Endocrinology*.

[19] Kawashima A., Yamazaki K., Hara T. (2013). Demonstration of innate immune responses in the thyroid gland: potential to sense danger and a possible trigger for autoimmune reactions. *Thyroid*.

[20] D'Ascola A., Scuruchi M., Ruggeri R. M. (2020). Hyaluronan oligosaccharides modulate inflammatory response, NIS and thyreoglobulin expression in human thyrocytes. *Archives of Biochemistry and Biophysics*.

[21] Guo Q., Wu Y., Hou Y. (2018). Cytokine secretion and pyroptosis of thyroid follicular cells mediated by enhanced NLRP3, NLRP1, NLRC4, and AIM2 inflammasomes are associated with autoimmune thyroiditis. *Front Immunol*.

[22] Li L., Yu H., Jiang Y. (2016). Genetic Variations of NLR family genes in Behcet's Disease. *Scientific Reports*.

[23] Shaw G. (2013). Polymorphism and single nucleotide polymorphisms (SNPs). *BJU international*.

[24] Meenakshi P., Ramya S., Shruthi T. (2013). Association of IL-1*β*+ 3954 C/T and IL-10-1082 G/A cytokine gene polymorphisms with susceptibility to tuberculosis. *Scandinavian journal of immunology*.

[25] Lee Y. H., Bae S. C. (2016). Association between functional NLRP3 polymorphisms and susceptibility to autoimmune and inflammatory diseases: a meta-analysis. *Lupus*.

[26] Wu Z., Wu S., Liang T. (2021). Association of NLRP3 rs 35829419 and rs 10754558 polymorphisms with risks of autoimmune diseases: a systematic review and meta-analysis. *Frontiers in genetics*.

[27] Zaaber I., Mestiri S., Hammedi H. (2016). Association of interleukin-1B and interleukin-4 gene variants with autoimmune thyroid diseases in Tunisian population. *Immunological investigations*.

[28] Suguna S. A., Nandal D. H., Kamble S. U., Bharatha A. M., Kunkulol R. A. (2014). Genomic DNA isolation from human whole blood samples by non enzymatic salting out method. *International Journal of Pharmacy and Pharmaceutical Sciences*.

[29] Wu H. C., Chang C. H., Ke H. L. (2011). Association of cyclooxygenase 2 polymorphic genotypes with prostate cancer in Taiwan. *Anticancer research*.

[30] Liontiris M. I., Mazokopakis E. E. (2017). A concise review of Hashimoto thyroiditis (HT) and the importance of iodine, selenium, vitamin D and gluten on the autoimmunity and dietary management of HT patients. Points that need more investigation. *Hellenic journal of nuclear medicine*.

[31] Thomsen H., Li X., Sundquist K., Sundquist J., Försti A., Hemminki K. (2020). Familial risks between Graves disease and Hashimoto thyroiditis and other autoimmune diseases in the population of Sweden. *Journal of Translational Autoimmunity*.

[32] Mikoś H., Mikoś M., Obara-Moszyńska M., Niedziela M. (2014). The role of the immune system and cytokines involved in the pathogenesis of autoimmune thyroid disease (AITD). *Endokrynologia Polska*.

[33] Zhao R., Zhou H., Su S. B. (2013). A critical role for interleukin-1*β* in the progression of autoimmune diseases. *International Immunopharmacology*.

[34] Shirodaria S., Smith J., IJ M. K., Kennett C. N., Hughes F. J. (2000). Polymorphisms in the IL-1A gene are correlated with levels of interleukin-1*α* protein in gingival crevicular fluid of teeth with severe periodontal disease. *Journal of dental research*.

[35] Pociot F., Mølvig J., Wogensen L., Worsaae H., JJEjoci N. (1992). A Taql polymorphism in the human interleukin-1*β* (IL-1*β*) gene correlates with IL-1*β* secretion in vitro. *European journal of clinical investigation*.

[36] Lacka K., Paradowska-Gorycka A., Maciejewski A., Kramer L., Herman W. A., Lacki J. K. (2014). Interleukin 1 beta (IL1beta) gene polymorphisms (SNP-511 and SNP+ 3953) in Hashimoto’s thyroiditis among the Polish population.

[37] Rashad N. M., Soliman M. H., Mousa M. M., Abd El-Fatah A. H. (2019). The influence of single-nucleotide polymorphisms of interleukin-1*β* -511 and +3954 on the susceptibility to Hashimoto’s thyroiditis in Egyptian women: immune-endocrine interactions. *The Egyptian Journal of Internal Medicine*.

[38] Chen R. H., Chen W. C., Chang C. T., Tsai C. H., Tsai F. J. (2005). Interleukin-1-beta gene, but not the interleukin-1 receptor antagonist gene, is associated with Graves' disease. *Journal of clinical laboratory analysis*.

[39] Liu Y. H., Chen R. H., Wu H. H. (2016). Association of interleukin-1*β* (IL1B) polymorphisms with Graves' ophthalmopathy in Taiwan Chinese patients. *Investigative Ophthalmology & Visual Science*.

[40] Wong K. H., Rong S. S., Chong K. K., Young A. L., Pang C. P., Chen L. J. (2015). Genetic associations of interleukin-related genes with Graves’ ophthalmopathy: a systematic review and meta-analysis. *Scientific Reports*.

[41] Liu J., Mao C., Dong L. (2019). Excessive iodine promotes pyroptosis of thyroid follicular epithelial cells in Hashimoto's thyroiditis through the ROS-NF-*κ*B-NLRP3 pathway. *Frontiers in endocrinology*.

[42] Zhang Q., Fan H. W., Zhang J. Z., Wang Y. M., Xing H. J. (2015). NLRP3 rs 35829419 polymorphism is associated with increased susceptibility to multiple diseases in humans. *Genetics and molecular research: GMR*.

[43] Yu P., Hao S., Zheng H., Zhao X., Li Y. (2018). Association of NLRP1 and NLRP3 polymorphisms with psoriasis vulgaris risk in the Chinese Han population. *Bio Med research international*.

[44] Sharon C., Jiquan S. (2020). Association of NLRP1 and NLRP3 gene polymorphism with psoriasis.

[45] Smith T. J., Jennings T. A., Sciaky D., Cao H. J. (1999). Prostaglandin-endoperoxide H synthase-2 expression in human thyroid epithelium. Evidence for constitutive expression in vivo and in cultured KAT-50 cells. *The Journal of biological chemistry*.

[46] Krawczyk-Rusiecka K., Wojciechowska-Durczynska K., Cyniak-Magierska A., Zygmunt A., Lewinski A. (2014). Assessment of cyclooxygenase-1 and 2 gene expression levels in chronic autoimmune thyroiditis, papillary thyroid carcinoma and nontoxic nodular goitre.

[47] Fuhrer D., Eszlinger M., Karger S. (2005). Evaluation of insulin-like growth factor II, cyclooxygenase-2, ets-1 and thyroid-specific thyroglobulin mRNA expression in benign and malignant thyroid tumours.

[48] Kim S. J., Lee J. H., Yoon J. S. (2003). Immunohistochemical expression of COX-2 in thyroid nodules. *The Korean journal of internal medicine*.

[49] Lee K. H., Kim H.-S., El-Sohemy A., Cornelis M. C., Uhm W.-S., Bae S.-C. (2006). Cyclooxygenase-2 genotype and rheumatoid arthritis. *The Korean journal of internal medicine*.

[50] Yun H.-R., Lee S.-O., Choi E. J., Shin H. D., Jun J.-B., Bae S.-C. (2008). Cyclooxygenase-2 polymorphisms and risk of rheumatoid arthritis in Koreans.

[51] Ucan B., Özbek M., Şahin M., Kizilgül M., Cakal E. (2017). Cyclooxygenase-2 (COX-2) gene polymorphism in patients withdifferentiated thyroid carcinomas in the Turkish population. *Turkish journal of medical sciences*.

